# Assessment of the performance of the Brazilian Portuguese Nottingham Health Profile in adult growth hormone deficiency and pulmonary hypertension

**DOI:** 10.12688/f1000research.27748.2

**Published:** 2021-08-10

**Authors:** Alice Heaney, Rafael W. R. de Oliveira, Mariana Bizzi, Ricardo Amorim Correa, Monica Corso Pereira, Suelem Simao Mol, Beatriz Santana Soares, Stephen P. McKenna, Antonio Ribeiro-Oliveira Jr, Study Investigators

**Affiliations:** 1 Galen Research Ltd, Manchester, UK; 2 Department of Internal Medicine, Federal University of Minas Gerais, Belo Horizonte, Minas Gerais, Brazil; 3 Department of Internal Medicine, State University of Campinas, Campinas, Sao Paulo, Brazil; 4 School of Health Sciences, University of Manchester, Manchester, UK

**Keywords:** health related quality of life, Nottingham Health Profile, adult growth hormone deficiency, pulmonary hypertension, CAMPHOR, QoL-AGHDA, patient-reported outcomes

## Abstract

**Background:** The Nottingham Health Profile (NHP) is a generic measure of perceived distress that has been used widely as an outcome measure in clinical practice and trials. The availability of two Brazilian datasets provided the opportunity to assess the psychometric performance of the NHP in different populations - adult growth hormone deficiency (GHD) and pulmonary hypertension (PH). The purpose of the study was to see how valuable the NHP could be in assessing outcomes in diseases where no disease-specific measures are available.

**Methods:** Secondary analyses were performed with NHP data. Patients diagnosed with adult GHD or PH were administered the NHP during clinic visits on two occasions, two weeks apart. A disease-specific measure of quality of life (QoL) was also administered to the relevant sample of patients on each occasion.

**Results:** The psychometric properties of the NHP were good for both disease groups. As expected, both samples reported high scores on energy level, the PH sample scored high on physical functioning and the GHD sample on emotional reactions. For both samples, most of the NHP sections were able to distinguish between groups of respondents with different ratings of perceived general health. While most sections of the NHP were relatively highly correlated with the QoL measures, pain and sleep did not seem to be important predictors of QoL in either of the samples.

**Conclusions:** The use of the NHP in adult GHD and PH populations in Brazil is not recommended as there are high-quality disease-specific measures available for each disease. However, where no disease-specific measures are available, the NHP can provide good descriptive information of the impact of disease on different patient populations.

## Introduction

The Nottingham Health Profile (NHP) is a generic measure of perceived distress that has been used widely as an outcome measure in clinical practice and trials. It consists of a profile of outcomes assessing different types of distress: physical mobility, pain, sleep, social isolation, emotional reactions, and energy level ^1^. Generic measures are considered valuable as they enable comparison of patient-reported outcomes between different populations and in assessments between healthy and unwell populations ^2^. The NHP has also been widely used as a comparator measure when validating disease-specific patient-reported outcome measures (PROMs). Newer, disease-specific PROMs have the advantage of asking questions that are more relevant to patients, while omitting questions that are less relevant.

As a result of the lack of responsiveness and age of the generic measures, they are being replaced by disease-specific PROMs. However, in medium and low-income countries, there is a lack of comprehensive information systems, creating methodological obstacles in evaluation studies and limiting the capacity to conduct longitudinal studies ^3^. Healthcare professionals, researchers and pharmaceutical companies are therefore reliant on generic outcome measures to collect information from patients because, theoretically, they can be used with any patient population. However, this means that outcome measures are selected based on availability rather than on quality.

The NHP has been used in two recent Brazilian studies. The measure was administered to patients with adult growth hormone deficiency (GHD) and pulmonary hypertension (PH) as comparator measures in the evaluation of Brazilian adaptations of the Quality of Life Assessment of Growth Hormone Deficiency in Adults (QoL-AGHDA) for GHD and the Cambridge Pulmonary Hypertension Outcome Review (CAMPHOR) for PH ^4, 5^. The availability of these data sets for secondary analysis provided the opportunity to assess the psychometric performance of the NHP in two different Brazilian populations.

Adult GHD results from decreased growth hormone secretion from the anterior pituitary gland. This is characterized by decreased lean body mass and increased fat mass, hyperlipidemia, cardiac dysfunction, decreased fibrinolysis and premature atherosclerosis, decreased muscle strength and exercise capacity, decreased bone mineral density, and decreased insulin resistance ^6^. The most frequent cause of childhood onset is idiopathic and may not necessarily be associated with other pituitary hormone deficiencies. In contrast, adult onset GHD results from hypothalamic-pituitary tumors and/or their treatment ^7^. GHD patients present with lower energy levels and more emotional problems than healthy individuals ^8^.

Pulmonary hypertension (PH) is a condition that may occur with a variety of disorders and is characterized by an increase in the pulmonary vascular resistance and in the mean pulmonary arterial pressure (mPAP). PH is currently classified into different groups according to similarities in pathophysiology and the presence of associated conditions ^9^. Group 1 (pulmonary arterial hypertension (PAH)) and Group 4 (PH due to pulmonary artery obstructions) are the most extensively studied and those for which there are approved drugs and/or procedures. Chronic thromboembolic pulmonary hypertension (CPTEH) is the most prevalent of the latter group. Patients with PH present with non-specific symptoms such as shortness of breath, progressive exertional dyspnea, chest pain, fatigue or syncope that progress over time, leading to right ventricular dysfunction and death ^10^. PH presents a significant impact on patients’ social and emotional well-being and on their daily life in general, with restrictions in the ability to perform everyday tasks ^11^.

The aim of this study was to investigate the psychometric properties of the NHP in adult GHD and PH populations. The purpose of the new analyses was to see how valuable the NHP could be in assessing outcomes in diseases where there is no effective disease-specific measure available.

## Methods

### Questionnaires

The NHP consist of 38 items with ‘yes’ and ‘no’ response alternatives, depending on whether that item fits the individual’s current situation. The possible score for each of the six sections ranges from zero to 100, with a higher score representing greater perceived distress. The NHP has been shown to have good reliability and validity as a generic measure ^12– 14^.

The QoL-AGHDA is the main measure of QoL in adults with GHD ^15^. The measure consists of 25 items with a dichotomous ‘Yes/No’ response format. A score of “1” is given to each item affirmed and these are summed to give a total score. A high score on the QoL-AGHDA indicates poor QoL. It was developed in parallel in the United Kingdom (UK), Sweden, Germany, Italy and Spain and has since been adapted into numerous additional languages.

The CAMPHOR was the first outcome measure developed specific to PH patients ^16^. This measure consists of two health-related quality of life (HRQL) scales (symptoms and activities) and a QoL scale. The CAMPHOR symptoms and QoL scales each consist of 25 items with a dichotomous response format. A total score is calculated by adding together the number of items affirmed, with a higher score indicating the presence of more symptoms or poorer QoL. The activities scale consists of 15 items that relate to activities described by patients as being affected by PH. Each item is scored according to the extent to which the patient rates themselves as being able to perform each activity, from zero (able to do on own without difficulty) to two (unable to do on own). Scores for each item are summed to give a total score ranging from zero to 30, with a higher score representing worse functioning. The CAMPHOR was originally developed and validated in the UK and has been adapted for use in many countries.

### Patients and data collection

Secondary data analyses were performed using NHP data that had been collected to assess the convergent validity of the Brazilian Portuguese QoL-AGHDA and CAMPHOR ^4, 5^ from December 2007 to October 2009 and from September 2018 to July 2019, respectively. Patients diagnosed with adult GHD or PAH/CTEPH - which will be called PH below - had completed the relevant questionnaires during clinic visits. The adult GHD patients had been diagnosed according to the international criteria ^17^ and were consecutively recruited from the following Brazilian endocrinology centres: Federal University of Minas Gerais, Hospital Santa Casa, Belo Horizonte (MG), University of Brasília, Brasília (DF), Federal University of Pernambuco, Recife (PE), SEMPR, the Federal University of Paraná, Curitiba (PR) and Hospital Brigadeiro, São Paulo (SP). These patients were not receiving GH therapy and replacement therapy for other pituitary deficiencies were stable for at least the six months before enrolment. The enrolled PH patients were consecutively recruited from the pulmonology centres at the Hospital das Clínicas, Federal University of Minas Gerais in Belo Horizonte, and Hospital das Clínicas of the University of Campinas, São Paulo. These participants were receiving PH-specific treatments ^18^. All patients were at least 18 years of age at enrolment.

For each patient sample, the NHP was administered on two occasions, two weeks apart. The QoL-AGHDA and CAMPHOR were also administered to the respective sample of patients on each occasion. Demographic and disease information were collected at both time points including age, gender, marital status, employment status, disease duration and perceived general health.

Permission to use the NHP, QoL-AGHDA and CAMPHOR was granted by the copyright holders of the three measures.

### Ethics

The study was approved by the Brazilian National Ethics Committee – Comite Nacional de Etica em Pesquisa (CONEP) and by the Local Ethics Committees from each participating institution for both adult GHD (CONEP 350/2008) and PH patients (CONEP 2857600). The study was conducted in accordance with the Helsinki declaration and all study participants provided written informed consent.

### Statistical analyses

Descriptive statistical analyses were conducted to examine the distributional properties of the NHP. The median and interquartile range were calculated due to the ordinal level of the data collected. The magnitude of floor and ceiling effects (% of patients scoring the minimum and maximum possible scores, respectively) were also assessed. A threshold of 15% was applied to indicate the presence of floor and ceiling effects ^19^.

The internal consistency of the NHP sections in adult GHD and PH samples was assessed through Cronbach’s alpha coefficients. These coefficients measure the extent to which the items in a scale are inter-related. A low alpha (below 0.7) indicates that the items do not work together to form a scale ^20^.

The test-retest reliability of the NHP sections was calculated as an estimate of their reproducibility over time when no change in condition is expected to have taken place. This was assessed by correlating scores obtained on the NHP sections on two different occasions using Spearman’s rank correlation coefficients. A minimum value of 0.85 is generally required to demonstrate that a PROM has low random measurement error ^21^.

Known group validity was assessed by testing the ability of the NHP sections to distinguish between groups of patients that differed by a known factor, considered likely to affect scores on the measure. The factor used for the present investigation was perceived general health (excellent / good / fair / poor / very poor). Non-parametric tests for independent samples (Mann-Whitney U-Test for two groups) were employed to test for differences in NHP section scores between groups. For this analysis, perceived general health was grouped into ‘Excellent / Good’ and ‘Fair / Poor / Very poor’. This was due to the small number of participants in the ‘Poor’ and ‘Very poor’ groups. Differences in NHP section scores according to demographic factors (age, gender) were also explored using the Mann-Whitney U-test. To produce groups of equal size, the sample was divided by the median age.

Total scores for each NHP section were correlated with total scores on the QoL-AGHDA and CAMPHOR QoL scale using Spearman’s rank correlation coefficients to explore which symptoms and functional limitations influence QoL in adult GHD and PH, respectively.

All statistical analyses were conducted using the Statistical Package for Social Sciences (SPSS) version 25.0. A value of p <.05 was considered statistically significant.

## Results

Demographic information for the GHD and PH samples is presented in Table 1. The GHD study population comprised primarily of patients with adult onset GHD. The PH sample consisted of patients who had been diagnosed as Group 1 PAH (n = 62) and Group 4 CTEPH (n = 40). Although the disease groups were not matched, the groups were similar in age. Similarly, both samples consisted of more females than males. Most of the respondents in both samples were married or living as married. However, considerably more GHD patients were employed and fewer were retired, compared to PH patients.

**Table 1.** Demographic details of adult growth hormone deficiency and pulmonary hypertension samples.

Variable	Growth hormone deficiency (n= 122)	Pulmonary hypertension * (n=102)
**Age, in years**		
Mean (SD)	46.1 (15.1)	48.8 (14.5)
Range	18 – 85	24.1 – 86.8
**Gender (n (%))**		
Male	46 (37.7)	20 (19.6)
Female	74 (60.7)	82 (80.4)
Missing	2 (1.6)	0 (0)
**Marital Status (n (%))**		
Married/Living as Married	69 (56.6)	66 (64.7)
Divorced	8 (6.6)	7 (6.9)
Widowed	5 (4.1)	5 (4.9)
Single	38 (31.1)	23 (22.5)
Missing	2 (1.6)	1 (1.0)
**Work Status (n (%))**		
Full-time	39 (32.0)	16 (15.7)
Part-time	4 (3.3)	3 (2.9)
Homemaker	24 (19.7)	17 (16.7)
Retired/retired due to disability	17 (13.9)	48 (47.1)
Long-term sick leave	12 (9.8)	3 (2.9)
Unemployed	12 (9.8)	8 (7.8)
Student	5 (4.1)	0 (0)
Other	7 (5.7)	6 (5.9)
Missing	2 (1.6)	1 (1.0)

* Includes Pulmonary Arterial Hypertension and Chronic thromboembolic pulmonary hypertension patients.

Descriptive statistics for the NHP sections are shown in Table 2. The distribution of scores for each section suggest that the patient groups experienced similar levels of distress. GHD patients reported marginally worse scores on the pain, emotional reactions and social isolation sections, compared to PH patients. However, PH patients scored higher on the physical mobility section. Major ceiling effects were observed for the energy section in both samples. Substantial floor effects were observed for all NHP sections with the exceptions of emotional reactions in the GHD sample and physical mobility in the PH sample. Raw NHP data for each participant are available as *Underlying data* ^22^.

**Table 2.** Descriptive statistics for NHP sections in adult Growth Hormone Deficiency and Pulmonary Hypertension samples.

	n	Median	Interquartile range	Min - Max	% scoring minimum	% scoring maximum
***Growth hormone deficiency***						
Energy	120	33.3	0 – 100	0 – 100	35.2	25.4
Pain	119	25	0 – 50	0 – 100	36.1	1.6
Emotional Reactions	118	33.3	11.1 – 66.7	0 – 100	13.9	3.3
Sleep	119	20	0 – 60	0 – 100	32.8	4.9
Social Isolation	121	20	0 – 60	0 – 100	35.2	7.4
Physical Mobility	119	25	0 – 37.5	0 – 87.5	27.9	0
Pulmonary hypertension *						
Energy	101	33.3	0 – 66.7	0 – 100	33.3	18.6
Pain	100	12.5	0 – 37.5	0 – 100	35.3	1
Emotional Reactions	101	22.2	11.1 – 66.7	0 – 100	18.6	3.9
Sleep	102	20	0 – 80	0 – 100	42.2	7.8
Social Isolation	102	0	0 – 40	0 – 100	57.8	6.9
Physical Mobility	101	37.5	12.5 – 62.5	0 – 87.5	10.8	0

* Includes Pulmonary Arterial Hypertension and Chronic thromboembolic pulmonary hypertension patients.

Cronbach’s alpha coefficients for the NHP sections are presented in Table 3. Coefficients above the minimum acceptable level of 0.7 were obtained for all sections in both samples. Spearman’s rank correlation coefficients between scores obtained on Time 1 and Time 2 for the NHP sections are shown in Table 4. All correlations were significant at the p<.01 level.

**Table 3.** Cronbach’s alpha coefficients for NHP sections in GHD (n=122) and PH (n=102) samples.

	Growth Hormone Deficiency	Pulmonary Hypertension *
Energy	0.78	0.74
Pain	0.84	0.81
Emotional Reactions	0.80	0.86
Sleep	0.76	0.84
Social Isolation	0.76	0.86
Physical Mobility	0.75	0.72

* Includes pulmonary arterial hypertension and chronic thromboembolic pulmonary hypertension patients.

**Table 4.** Reproducibility (Spearman’s rank correlation coefficients) of NHP sections in adult Growth Hormone Deficiency and Pulmonary Hypertension samples.

		Growth hormone deficiency		Pulmonary hypertension *
	n		n	
Energy	78	0.83	94	0.81
Pain	80	0.86	93	0.81
Emotional Reactions	75	0.85	94	0.91
Sleep	80	0.82	95	0.85
Social Isolation	82	0.76	95	0.85
Physical Mobility	78	0.78	94	0.91

* Includes pulmonary arterial hypertension and chronic thromboembolic pulmonary hypertension patients.

Disease duration and perceived general health are presented in Table 5. A Mann-Whitney U-test revealed that the PH sample had significantly worse ratings of general health than the GHD sample (U = 5070.5, p < .05).

**Table 5.** Disease duration and perceived general health of adult Growth Hormone Deficiency and Pulmonary Hypertension samples.

	Growth Hormone Deficiency (n= 122)	Pulmonary Hypertension * (n=102)
**Disease duration, in years**		
Mean (SD)	11.7 (10.7)	7.6 (6.3)
Range	1 - 40	0.33 - 44
**Perceived general health (n (%))**		
Excellent	12 (9.8)	4 (3.9)
Good	46 (37.7)	38 (37.3)
Fair	55 (45.1)	38 (37.3)
Poor/Very poor	7 (5.7)	21 (20.6)
Missing	2 (1.6)	1 (1.0)

* Includes pulmonary arterial hypertension and chronic thromboembolic pulmonary hypertension patients.

Table 6a and Table 6b show NHP section scores grouped by perceived general health in the GHD and PH samples, respectively. For both samples, most of the NHP sections were able to distinguish between groups of respondents that differed according to their perceived general health. For these NHP sections, individuals who considered their general health to be ‘Excellent / Good’ had significantly better scores on the NHP sections than those who rated their health less favourably. The exceptions were the sleep section in the GHD sample and the social isolation section in the PH sample.

**Table 6a.** Median NHP section scores by perceived general health in adult Growth Hormone Deficiency patients.

	Energy level	Pain	Emotional reactions	Sleep	Social isolation	Physical mobility
	Median (IQR)	Median (IQR)	Median (IQR)	Median (IQR)	Median (IQR)	Median (IQR)
**General Health**						
Excellent/Good	33.3 (0 – 66.7)	12.5 (0 – 37.5)	22.2 (2.8 – 44.4)	20 (0 – 60)	20 (0 – 40)	12.5 (0 – 25)
Fair/Poor/Very poor	66.7 (8.3 – 100)	25 (0 – 62.5)	44.4 (22.2 – 66.7)	40 (0 – 60)	20 (20 – 80)	25 (12.5 – 50)
***p***	<.01	<.05	<.01	.33	<.01	<.05

**Table 6b.** Median NHP section scores by perceived general health in pulmonary hypertension * patients.

	Energy level	Pain	Emotional reactions	Sleep	Social isolation	Physical mobility
	Median (IQR)	Median (IQR)	Median (IQR)	Median (IQR)	Median (IQR)	Median (IQR)
**General Health**						
Excellent/Good	0 (0 – 33.3)	0 (0 – 25)	11.1 (0 – 25)	0 (0 – 45)	0 (0 – 20)	25 (0 – 37.5)
Fair/Poor	66.7 (33.3 – 100)	25 (12.5 – 50)	44.4 (22.2 – 66.7)	40 (0 – 80)	0 (0 – 60)	50 (25 – 62.5)
***p***	< .001	<.001	<.001	<.01	.07	<.001

* Includes Pulmonary Arterial Hypertension and Chronic thromboembolic pulmonary hypertension patients.

Table 7 shows NHP section scores by age group (above versus below median age) and gender in the adult GHD and PH sample combined. A Mann Whitney U-test found no significant difference in scores between older and younger patients on any of the NHP sections. Significant differences in scores were found between females and males. Females reported significantly higher scores than males on all sections except energy level and social isolation.

**Table 7.** Median NHP section scores by age and gender in combined sample of adult growth hormone deficiency and pulmonary hypertension * patients.

	Energy level	Pain	Emotional reactions	Sleep	Social isolation	Physical mobility
	Median (IQR)	Median (IQR)	Median (IQR)	Median (IQR)	Median (IQR)	Median (IQR)
Age **						
Below median	33.3 (0 – 66.7)	12.5 (0 – 50)	33.3 (11.1 – 66.7)	20 (0 – 60)	20 (0-60)	25 (12.5 – 50)
Above median	33.3 (0 – 66.7)	18.8 (0 – 50)	22.2 (11.1 – 55.6)	20 (0 – 60)	0 (0 – 20)	25 (12.5 – 43.8)
***P***	.39	.29	.17	.87	.05	.83
**Gender**						
Male	33.3 (0 – 66.7)	12.5 (0 – 31.3)	22.2 (11.1 – 44.4)	20 (0 – 40)	20 (0 – 40)	25 (0 – 37.5)
Female	33.3 (0 – 83.3)	25 (0 – 50)	33.3 (11.1 – 66.7)	40 (0 – 80)	20 (0 – 60)	25 (12.5 – 50)
***P***	.24	<.05	<.05	<.05	.88	<.05

* Includes pulmonary arterial hypertension and chronic thromboembolic pulmonary hypertension patients. ** Sample divided by median age of 46.3.

**Figure 1.**
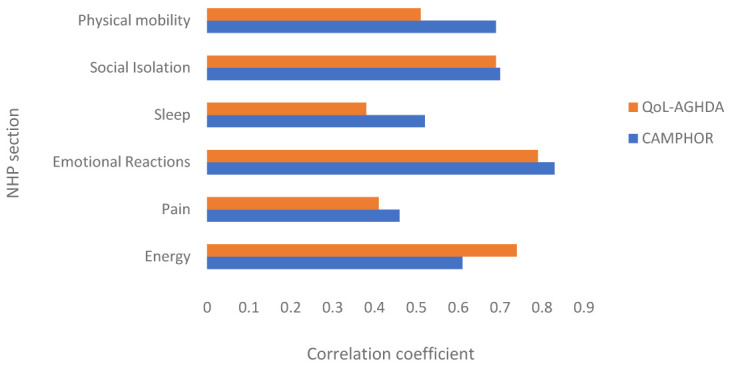
Spearman’s rank correlation coefficients between NHP section scores and total scores on the QoL-AGHDA and CAMPHOR QoL scale. Note: All correlations were significant at p<.01.

Figure 1 shows the Spearman’s rank correlations between scores on the NHP sections and disease-specific measures of QoL in patients with GHD (QoL-AGHDA) and PH (CAMPHOR QoL scale). Total scores on the QoL-AGHDA correlated most highly with the emotional reactions and energy level sections. The CAMPHOR QoL scale correlated relatively highly with the emotional reactions and social isolation sections but was also influenced by problems with physical mobility. A weak association was observed between total scores on the QoL-AGHDA and the sleep section of the NHP. Scores on the pain section of the NHP did not correlate highly with QoL scores on either the QoL-AGHDA or the CAMPHOR.

## Discussion

The use of patient-reported outcomes to evaluate different interventions is becoming commonplace in clinical studies and trials. For many years, generic outcome measures such as the SF-36 and NHP were used for this purpose. However, these generic measures have been shown to lack sensitivity to change over time. In addition, all generic measures lack the responsiveness to be fully effective in clinical trials because their items are intended to be suitable for all possible illnesses. This means that they miss important aspects of the disease and include irrelevant items ^23– 25^. Consequently, there has been a move towards the use of disease-specific outcome measures which are able to ask more relevant questions and to measure outcome more accurately. Many researchers also continue to use the generic outcome measures together with disease-specific measures. While disease-specific measures are becoming more widely available, they have yet to be widely adapted for use in medium- and low-income countries. At the same time, clinical trials are being more frequently undertaken in such countries. Such studies require patient-reported outcome measures.

The current study looked at the performance of the generic NHP in two patient groups in Brazil, adult GHD and PH. NHP data were available from these patients as the measure had been used to help validate new disease-specific outcome measures. Analyses were undertaken to see whether the NHP would be a useful outcome measure for trials in other diseases.

The two populations had comparable ages. However, a greater proportion of PH patients were retired. This difference could be explained by the PH sample experiencing poorer general health, compared to the GHD sample. NHP section scores in both groups were strongly related to perceived general health.

Scores on the NHP sections were not influenced by age but women scored higher (had worse health status) than men. These findings are consistent with previous research reporting gender differences in QoL impairments for both patient populations studied ^26, 27^.

Overall, the psychometric properties of the NHP sections were good for both disease populations. Estimates of internal consistency suggest that the items in the six sections of the NHP are sufficiently inter-related to form scales. Test-retest reliability (reproducibility) was better than that achieved by other generic measures though slightly lower than ideal. While only pain and emotional reactions demonstrated test-retest reliability coefficients of 0.85 and above in the GHD sample, all NHP sections in both patient groups were above the usually quoted acceptable value of 0.7 ^28, 29^.

A large proportion of GHD and PH patients obtained the lowest possible score on most of the NHP sections. This indicates that the measure is not well targeted to these samples, which could be explained by the relatively mild perceived general health of the GHD and PH patients. Scores on the NHP confirmed the presence of common problems experienced by patients. Both samples reported high scores on energy level, the PH sample scored high on physical functioning and the GHD sample on emotional reactions. The correlations between scores obtained on the disease-specific measures of QoL and the NHP sections suggests that pain and sleep did not seem to be important predictors of QoL in either of the samples.

A limitation of the current study is the lack of clinical information about the patient groups. It must be noted that the data collected for disease duration in the adult GHD sample should be interpreted with caution. The time between initial onset of GHD and diagnosis is likely to affect disease severity. In addition, the lack of inclusion of patients with GHD of childhood onset or GH-treated patients is a weakness, although relatively few GHD patients in Brazil are currently prescribed replacement growth hormone. It would have been valuable to collect WHO functional class information for the PH patients to explore whether the NHP could detect differences in health status relating to objective disease severity. 

The use of the NHP in adult GHD and PH populations in Brazil is not recommended as there are high-quality disease-specific measures available for each disease. However, where no disease-specific measures are available, the NHP can provide good descriptive information of the impact of disease on different patient populations.

## Data availability

### Underlying data

 Figshare: Copy of Raw data.xlsx. https://doi.org/10.6084/m9.figshare.13299701.v1 ^22^.

This file contains de-identified patient-reported-outcome raw data from NHP questionnaires.

Data are available under the terms of the Creative Commons Attribution 4.0 International license (CC-BY 4.0).
