# Supplementary material for: Assessment of the performance of the Brazilian Portuguese Nottingham Health Profile in adult growth hormone deficiency and pulmonary hypertension
Source: F1000Res. 2021 Aug 10;9:1399. Originally published 2020 Dec 4. [Version 2] doi: 10.12688/f1000research.27748.2 (PMC8447051; doi:10.12688/f1000research.27748.2)
Supplement: Peer review history

# Peer review: Reviewer response for version 2

**DOI:** 10.5256/f1000research.59289.r91599

**Published:** 2021-09-16

**Authors:** Jaquelina Sonoe Ota-Arakaki

**Affiliations:** 1 Division of Respiratory Diseases, Federal University of São Paulo (UNIFESP), São Paulo, Brazil

**Dear Authors**

**Follow my report of review V2**

 Dear Reviewer,

 Thank you for your time dedicated to this manuscript.

 Please find below the answers to your comments.

**The authors present the secondary data analyses of the Nottingham Health Profile (NHP), a generic measure of perceived distress, that had been collected to assess the validity of the Brazilian Portuguese QoL-AGHDA and CAMPHOR in a sample of adult GHD and PH in Brazil and previously published in two articles.**

**Despite the demonstration of high scores on energy level, functional and emotional reactions in GHD or PH patients in the studied sample, the authors concluded that the NHP should not be recommended in the presence the validating disease-specific patient-reported outcome measures. However, the aim of the study to assess the role of the NHP in outcomes in GHD and PH, has not been evaluated.**

 First of all, thank you for the comment provided. The aim of the study was to assess the psychometric performance of the NHP in different patient populations. This was achieved through examining the reliability and validity of the measure. The results indicated that its psychometric properties are adequate but the NHP has some psychometric weaknesses. Consequently, it is only recommended in instances where there is no disease-specific measure available.

**“ *The purpose of the new analyses was to see how valuable the NHP could be in assessing outcomes in diseases where there is no effective disease-specific measure available.”***

**In Pulmonary vascular diseases and others, we usually use the term “assessing outcomes” the meaning to assess probability of outcomes such as hospitalization and mortality. Please, make the text clear.**

**Minor comments:**

**1. Although the authors report in the introduction that the CTEPH is more prevalent, in this sample there is a greater prevalence of PAH.**

 We think this reviewer has not got it correctly. The introduction states that CTEPH is the most prevalent form of PH due to pulmonary artery obstructions (Group 4). It does not state that CTEPH is more prevalent than PAH in our sample.

**Dear authors, you are correct. I misunderstood the first reading when I had understood that the CTEPH would be more prevalent than PAH.**

**2.** **Describe the patient’s inclusion period and whether it was consecutive.**

 Thank you for flagging this. The present study is a re-analysis of a database of previous research from our team. The patients were consecutively admitted to the respective clinics and those who agreed to participate were then included. We have amended the manuscript accordingly in the methods section to include the items that have been pointed by this reviewer. 

**Ok.**

**3. In the sample there is missing demographic data (n=2) raising the fragility of data of these patients. I suggest excluding them from the analyses.**

 That's an interesting point which have discussed further, and we believe it would be unfair to remove the data of these individuals from the analyses considering they did not fail to provide responses to the questionnaire. They would be omitted where demographic variables were being tested.

**I agree that the statistic analyses were not compromised where demographic variables were not being tested. Still, the missing demographic data of these two patients demonstrates weakness of all information about them.**

**4.** **The follow-up for a 44 year in PH is unusual. Justify or remove it from the analysis (out-liner).**

 While we acknowledge that a 44 year follow-up in PH is quite unusual, this patient was 55yo and had developed PH as consequence of congenital cardiopathy diagnosed in his childhood.

**The results of the study support the conclusion that:**

**“Overall, the psychometric properties of the NHP sections were good for both disease populations”**

**The paragraph below can be presented as the authors' opinion. The comparison results with the specific questionnaires were presented in two previous studies by the authors with the same cohorts of patients.**

**"The use of the NHP in adult GHD and PH populations in Brazil is not recommended as there are high-quality disease-specific measures available for each disease. However, where no disease-specific measures are available, the NHP can provide good descriptive information of the impact of disease on different patient populations."**

Is the work clearly and accurately presented and does it cite the current literature?

Partly

If applicable, is the statistical analysis and its interpretation appropriate?

Partly

Are all the source data underlying the results available to ensure full reproducibility?

Yes

Is the study design appropriate and is the work technically sound?

Partly

Are the conclusions drawn adequately supported by the results?

Partly

Are sufficient details of methods and analysis provided to allow replication by others?

Yes

Reviewer Expertise:

Pulmonary Circulation Diseases

I confirm that I have read this submission and believe that I have an appropriate level of expertise to confirm that it is of an acceptable scientific standard.

# Peer review: Reviewer response for version 2

**DOI:** 10.5256/f1000research.59289.r91600

**Published:** 2021-08-23

**Authors:** Günter K. Stalla, Ulrich Renner

**Affiliations:** 1 Medizinische Klinik und Poliklinik IV, Klinikum der Universität, Ludwig-Maximilians Universität München, Munich, Germany; 2 Department of Internal Medicine, Endocrinology and Clinical Chemistry, Max-Planck Institute of Psychiatry, Munich, Germany

The paper is now suitable for indexing.

Is the work clearly and accurately presented and does it cite the current literature?

Yes

If applicable, is the statistical analysis and its interpretation appropriate?

Yes

Are all the source data underlying the results available to ensure full reproducibility?

Yes

Is the study design appropriate and is the work technically sound?

Yes

Are the conclusions drawn adequately supported by the results?

Yes

Are sufficient details of methods and analysis provided to allow replication by others?

Yes

Reviewer Expertise:

Endocrine disorders

We confirm that we have read this submission and believe that we have an appropriate level of expertise to confirm that it is of an acceptable scientific standard.

# Peer review: Reviewer response for version 1

**DOI:** 10.5256/f1000research.30682.r78848

**Published:** 2021-03-18

**Authors:** Günter K. Stalla, Ulrich Renner

**Affiliations:** 1 Medizinische Klinik und Poliklinik IV, Klinikum der Universität, Ludwig-Maximilians Universität München, Munich, Germany; 2 Department of Internal Medicine, Endocrinology and Clinical Chemistry, Max-Planck Institute of Psychiatry, Munich, Germany

Heany and colleagues have studied in two cohorts of patients with adult growth hormone deficiency (GHD) or pulmonary hypertension (PH) whether the Nottingham Health Profile (NHP), a generic measure of perceived distress, gives similar information as specific tests for quality of life (QoL) such as the QoL-AGHDA (for patients with GHD) or CAMPHOR (for patients with PH). They found that the NHP provides good information about the disease for the two groups of patients and may therefore an option in countries with less well developed health care system.

 However, when possible the disease-specific tests should be used as they predict more reliably state and outcome of the corresponding disease. In general, the study is well done and the results are important and interesting.

**Minor comment**

 I'm wondering why the authors have not excluded patients, for which demographic data are missing (table 2), from the study.

Is the work clearly and accurately presented and does it cite the current literature?

Yes

If applicable, is the statistical analysis and its interpretation appropriate?

Yes

Are all the source data underlying the results available to ensure full reproducibility?

Yes

Is the study design appropriate and is the work technically sound?

Yes

Are the conclusions drawn adequately supported by the results?

Yes

Are sufficient details of methods and analysis provided to allow replication by others?

Yes

Reviewer Expertise:

Endocrine disorders

We confirm that we have read this submission and believe that we have an appropriate level of expertise to confirm that it is of an acceptable scientific standard.

# Peer review: Reviewer response for version 1

**DOI:** 10.5256/f1000research.30682.r77612

**Published:** 2021-03-04

**Authors:** Jaquelina Sonoe Ota-Arakaki

**Affiliations:** 1 Division of Respiratory Diseases, Federal University of São Paulo (UNIFESP), São Paulo, Brazil

The authors present the secondary data analyses of the Nottingham Health Profile (NHP), a generic measure of perceived distress, that had been collected to assess the validity of the Brazilian Portuguese QoL-AGHDA and CAMPHOR in a sample of adult GHD and PH in Brazil and previously published in two articles.

 Despite the demonstration of high scores on energy level, functional and emotional reactions in GHD or PH patients in the studied sample, the authors concluded that the NHP should not be recommended in the presence the validating disease-specific patient-reported outcome measures. However, the aim of the study to assess the role of the NHP in outcomes in GHD and PH, has not been evaluated.

**Minor comments:**

1. Although the authors report in the introduction that the CTEPH is more prevalent, in this sample there is a greater prevalence of PAH.
2. Describe the patient’s inclusion period and whether it was consecutive.
3. In the sample there is missing demographic data (n=2) raising the fragility of data of these patients. I suggest excluding them from the analyses.
4. The follow-up for a 44 year in PH is unusual. Justify or remove it from the analysis (out-liner).

Is the work clearly and accurately presented and does it cite the current literature?

Partly

If applicable, is the statistical analysis and its interpretation appropriate?

Partly

Are all the source data underlying the results available to ensure full reproducibility?

Yes

Is the study design appropriate and is the work technically sound?

Partly

Are the conclusions drawn adequately supported by the results?

Partly

Are sufficient details of methods and analysis provided to allow replication by others?

Yes

Reviewer Expertise:

Pulmonary Circulation Diseases

I confirm that I have read this submission and believe that I have an appropriate level of expertise to confirm that it is of an acceptable scientific standard, however I have significant reservations, as outlined above.

# Response

**Published:** 2021-08-05

**Authors:** Antonio Ribeiro-Oliveira Jr

**Affiliations:** Federal University of Minas Gerais, Brazil

Dear Reviewer,

 Thank you for your time dedicated to this manuscript.

 Please find below the answers to your comments.

**The authors present the secondary data analyses of the Nottingham Health Profile (NHP), a generic measure of perceived distress, that had been collected to assess the validity of the Brazilian Portuguese QoL-AGHDA and CAMPHOR in a sample of adult GHD and PH in Brazil and previously published in two articles.**

**Despite the demonstration of high scores on energy level, functional and emotional reactions in GHD or PH patients in the studied sample, the authors concluded that the NHP should not be recommended in the presence the validating disease-specific patient-reported outcome measures. However, the aim of the study to assess the role of the NHP in outcomes in GHD and PH, has not been evaluated.**

 First of all, thank you for the comment provided. The aim of the study was to assess the psychometric performance of the NHP in different patient populations. This was achieved through examining the reliability and validity of the measure. The results indicated that its psychometric properties are adequate but the NHP has some psychometric weaknesses. Consequently, it is only recommended in instances where there is no disease-specific measure available.

**Minor comments:**

**1. Although the authors report in the introduction that the CTEPH is more prevalent, in this sample there is a greater prevalence of PAH.**

 We think this reviewer has not got it correctly. The introduction states that CTEPH is the most prevalent form of PH due to pulmonary artery obstructions (Group 4). It does not state that CTEPH is more prevalent than PAH in our sample.

**2.** **Describe the patient’s inclusion period and whether it was consecutive.**

 Thank you for flagging this. The present study is a re-analysis of a database of previous research from our team. The patients were consecutively admitted to the respective clinics and those who agreed to participate were then included. We have amended the manuscript accordingly in the methods section to include the items that have been pointed by this reviewer. 

**3. In the sample there is missing demographic data (n=2) raising the fragility of data of these patients. I suggest excluding them from the analyses.**

 That's an interesting point which have discussed further, and we believe it would be unfair to remove the data of these individuals from the analyses considering they did not fail to provide responses to the questionnaire. They would be omitted where demographic variables were being tested.

 4. **The follow-up for a 44 year in PH is unusual. Justify or remove it from the analysis (out-liner).**

 While we acknowledge that a 44 year follow-up in PH is quite unusual, this patient was 55yo and had developed PH as consequence of congenital cardiopathy diagnosed in his childhood.
